# Potential Vitamin B6 Depletion During Carbidopa/Levodopa Therapy in Parkinson's Disease: A Scoping Review of Direct Evidence

**DOI:** 10.7759/cureus.114123

**Published:** 2026-08-07

**Authors:** Patryk Sielaff, Monika Lis, Jan Pawłasek, Rafał R Koch, Mateusz Sucharski, Barbara Michałowicz, Patryk Cierpiał, Magdalena Puziuk, Paweł Mikołajczak, Albert Staranowicz, Martyna Dziurzyńska, Patryk Dziurzyński, Magdalena Kiełbasiewicz, Hubert Piwar

**Affiliations:** 1 Faculty of Medical Sciences, Warsaw Medical Academy of Applied Sciences, Warsaw, POL; 2 Department of Medicine, Masovian Bródnowski Hospital, Warsaw, POL; 3 Department of Pharmacotherapy and Pharmaceutical Care, Medical University of Warsaw, Warsaw, POL; 4 Department of General Practice, Military Institute of Medicine-National Research Institute, Warsaw, POL; 5 Department of Medicine, Powiatowy Szpital im. Władysława Biegańskiego w Iławie, Iława, POL; 6 Department of Medicine, Central Clinical Hospital in Warsaw, Warsaw, POL; 7 Department of Medicine, Dr. Aleksander Majkowski District Hospital, Kartuzy, POL; 8 Department of Medicine, National Medical Institute of the Ministry of the Interior and Administration, Warsaw, POL; 9 Department of Orthopaedics and Traumatology of the Musculoskeletal System, Medical University of Warsaw, Warsaw, POL; 10 Department of Internal Medicine and Gastroenterology, Masovian Bródnowski Hospital, Warsaw, POL; 11 Department of Gastroenterology, Bielanski Hospital, Warsaw, POL; 12 Department of Medicine, Nowodworskie Centrum Medyczne, Nowy Dwór Mazowiecki, POL; 13 Department of Medicine, Dr. Anna Gostynska Wolski Hospital, Warsaw, POL

**Keywords:** levodopa-carbidopa, levodopa/carbidopa intestinal gel infusion, parkinson’s disease (pd), pyridoxal phosphate, vitamin b6 deficiency

## Abstract

Vitamin B6 depletion during carbidopa/levodopa therapy has been linked to polyneuropathy, seizures, and anemia, but the direct evidence is heterogeneous, and causality remains uncertain. We conducted a focused scoping review of direct empirical evidence on vitamin B6 biology during carbidopa/levodopa exposure and on vitamin B6-related clinical consequences supported by biochemical assessment. We also included studies of biomarker-guided monitoring, supplementation, and direct carbidopa-vitamin B6 chemistry. PubMed, Scopus, and Web of Science were searched without date or language limits on July 21, 2026. Of 656 database records, 448 unique records were screened, 161 reports were assessed in full, and 36 reports met the focused criteria. These comprised 34 reports involving human participants, including 18 group-level studies and 16 case reports or series, and two nonclinical experimental reports. Direct chemistry supports stable carbidopa-pyridoxal 5'-phosphate binding, and high-dose carbidopa exposure in dogs produced a preventable deficiency phenotype. Human studies reported lower vitamin B6 biomarkers most consistently with high or rapidly increased exposure, but findings were not uniform, and dose, route, advanced disease, nutrition, supplementation, and overlapping cohorts could not be separated. Severe deficiency accompanied axonal neuropathy, refractory seizures, and microcytic or sideroblastic-pattern anemia in individual case reports. No study established population-level incidence of vitamin B6 depletion or attributable clinical outcomes, a causal exposure threshold, an optimal testing interval, or a prophylactic dose. Direct vitamin B6 testing, interpreted with laboratory-specific reference intervals and complementary nutritional, hematologic, and neurologic assessment, is better supported by the available evidence than reliance on homocysteine alone. Prospective exposure-resolved studies are required before route-specific risk or universal supplementation can be recommended.

## Introduction and background

Parkinson's disease is a progressive neurodegenerative disorder characterized by bradykinesia, rigidity, resting tremor, and a broad nonmotor burden that can include cognitive, sleep, psychiatric, autonomic, and sensory disturbances. More than 8.5 million people were estimated to be living with Parkinson's disease worldwide in 2019 [1]. Levodopa is recommended when early motor symptoms impair quality of life and remains the most effective symptomatic therapy across the disease course. Oral carbidopa/levodopa is the usual starting route; continuous intestinal or subcutaneous delivery may be used in selected patients with advanced levodopa-responsive disease and disabling motor fluctuations despite optimized less-invasive treatment [2].

Levodopa improves motor symptoms and activities of daily living but does not slow the underlying neurodegenerative process [1,2]. Immediate-release carbidopa/levodopa begins releasing drug within 30 minutes, and carbidopa extends the plasma half-life of levodopa to approximately 1.5 hours. The duration of clinical benefit from an individual dose varies and may shorten as end-of-dose wearing-off develops. Dyskinesia and nausea are the most commonly reported adverse reactions; orthostatic hypotension, somnolence, hallucinations, confusion, and motor fluctuations are also clinically important [3]. A systematic review estimated peripheral neuropathy in 30.2% of patients receiving oral levodopa and 42.1% of those receiving levodopa/carbidopa intestinal gel; however, heterogeneous definitions and study designs mean that these values neither estimate vitamin B6-deficiency-attributable neuropathy nor prove treatment causation [4]. Comparable incidence estimates for vitamin B6-attributable seizures or anemia are unavailable.

Carbidopa inhibits peripheral aromatic L-amino acid decarboxylase, increases levodopa delivery to the brain, and reduces peripheral dopamine-related adverse effects. Pyridoxal 5'-phosphate is the active coenzyme form of vitamin B6 and is required by aromatic L-amino acid decarboxylase and many other enzymes involved in amino acid metabolism, neurotransmitter synthesis, transsulfuration, and heme synthesis. Contemporary structural and kinetic work shows that carbidopa inhibits human dopa decarboxylase through a stable pyridoxal 5'-phosphate hydrazone [5]. In dogs, high-dose carbidopa produced low circulating pyridoxine, abnormal functional vitamin B6 markers, and neuropathic pathology that was prevented by pyridoxine coadministration [6]. These experiments establish biological plausibility, but target binding and high-dose toxicity in dogs do not establish clinically important whole-body vitamin B6 depletion during therapeutic human exposure.

Direct human evidence is mixed. A controlled cross-sectional study found lower plasma pyridoxal 5'-phosphate and higher homocysteine in levodopa/carbidopa-treated patients than in levodopa-naive patients with Parkinson's disease [7]. Small oral and intestinal cohorts subsequently reported frequent low vitamin B6 values and inverse associations with levodopa/carbidopa dose [8,9]. Long-term levodopa/carbidopa intestinal gel studies captured decreased vitamin B6 as a laboratory or adverse event finding, but monitoring began late in some protocols, and supplementation was common, obscuring untreated trajectories [10]. More recently, vitamin B6 decreased within approximately eight days of starting continuous subcutaneous foslevodopa/foscarbidopa [11], and serial case observations linked the same formulation to biochemical deterioration and neuropathy [12]. Collectively, these observations suggest an exposure-related signal across routes, while leaving the independent contributions of carbidopa, levodopa, formulation, disease severity, and nutritional reserve unresolved.

The most consequential reports describe severe but selected clinical presentations. Profound deficiency occurred with refractory seizures or status epilepticus during high-dose oral or intestinal therapy [13,14], with axonal neuropathy and other metabolic abnormalities during intestinal or subcutaneous infusion [15-17], and with severe microcytic or sideroblastic-pattern anemia [18,19]. Treatment responses support clinical plausibility, but many patients also had weight loss, poor intake, folate or vitamin B12 abnormalities, diabetes, renal disease, or other competing causes. Case reports cannot estimate incidence, and biomarker normalization does not prove that vitamin B6 deficiency was the only cause of neurologic injury.

The evidence spans molecular experiments, animal toxicology, cross-sectional biomarker studies, uncontrolled treatment cohorts, overlapping extension studies, and case reports. Its heterogeneity makes evidence mapping more appropriate than statistical pooling. The objective of this focused scoping review was to characterize only the direct empirical evidence linking carbidopa/levodopa exposure to altered vitamin B6 biology, clinical consequences, or biomarker-guided management; distinguish positive from negative findings; and identify what can and cannot currently support monitoring in clinical practice.

## Review

Methods

Design and Reporting Framework

This focused scoping review was reported in accordance with the Preferred Reporting Items for Systematic Reviews and Meta-Analyses extension for Scoping Reviews (PRISMA-ScR) [20]. A scoping design was retained because the question encompassed human and nonclinical evidence, multiple formulations, heterogeneous vitamin B6 measures, several clinical phenotypes, and study designs that could not support a common effect estimate.

This review is a focused secondary narrowing of a broader evidence map. The original search and initial screening addressed direct, supporting, and contextual evidence. The present direct-evidence eligibility rule was finalized after that broad full-text mapping but before the focused synthesis. The focused criteria were then reapplied to every retrieved full report. This post hoc narrowing is reported as a methodological amendment from the original broader review plan and is considered in the limitations. No review protocol was prospectively registered in the Open Science Framework or another registry, and no protocol was published.

Eligibility Criteria

The clinical population comprised people with Parkinson's disease who received oral levodopa/carbidopa, levodopa/carbidopa intestinal gel, levodopa/entacapone/carbidopa intestinal gel, or foslevodopa/foscarbidopa. Nonclinical systems were eligible only when an experiment directly tested carbidopa binding to pyridoxal 5'-phosphate, an effect of carbidopa on vitamin B6 status, or prevention of a carbidopa-associated deficiency phenotype.

The concept was direct empirical evidence of altered vitamin B6 biology or its management. A human report qualified if it met at least one of four content-based conditions. First, it measured a vitamin B6 vitamer, pyridoxal 5'-phosphate, or a clearly identified functional vitamin B6 marker during explicit carbidopa-containing exposure. Second, it prospectively or retrospectively assessed decreased vitamin B6 as an outcome or adverse event. Third, it documented a clinical consequence explicitly linked to measured vitamin B6 deficiency. Fourth, it evaluated vitamin B6-directed monitoring or supplementation using measured vitamin B6 status. Negative and neutral results remained eligible. Homocysteine, neuropathy, seizures, and anemia were not accepted as vitamin B6 evidence unless the report explicitly assessed or linked them to vitamin B6 biology.

Eligible designs included controlled and uncontrolled clinical studies, observational cohorts, open-label extensions, post hoc safety analyses, case reports and series, preprints with sufficient empirical detail, and direct nonclinical experiments. Reviews, guidelines, editorials, historical pharmacology without a depletion outcome, levodopa-only or benserazide-only evidence that could not inform an explicit carbidopa exposure, and mechanistic reports without a direct carbidopa-vitamin B6 experiment were excluded from the focused evidence set. Retracted reports were excluded. No date or language filter was imposed. A non-English report required an adequate English abstract, a supplied translation, or an interpretable full report. Records without abstract text proceeded to full-text assessment only if a usable abstract or the corresponding full report was subsequently obtained; otherwise, they were excluded during record screening because relevance could not be assessed.

Information Sources and Search

PubMed, Scopus, and Web of Science Core Collection were searched on July 21, 2026. The strategies combined controlled vocabulary where available and text terms for Parkinson's disease, levodopa or carbidopa formulations, and vitamin B6 or pyridoxal 5'-phosphate. Scopus and Web of Science also used an outcome block covering deficiency, depletion, neuropathy, seizures, anemia, homocysteine, monitoring, and supplementation. No date or language limits were applied. The searches yielded 135 PubMed records, 419 Scopus records, and 102 Web of Science records, for 656 records before deduplication. The World Health Organization (WHO) disease-burden fact sheet [1], National Institute for Health and Care Excellence (NICE) treatment guideline [2], current SINEMET (carbidopa/levodopa) prescribing information [3], the 2019 systematic review of levodopa-associated neuropathy [4], and the March 20, 2026, United States Food and Drug Administration (FDA) Drug Safety Communication [21] were identified outside the database evidence set and used only for background or regulatory context. They were not screened, charted, or counted among the 36 included reports. The exact database search strategies are presented in Appendix A.

Record Management and Selection

Search results were imported into EndNote™ (Clarivate, Philadelphia, PA, USA) and consolidated in a single review library. Deduplication in EndNote used digital object identifiers, PubMed identifiers, and title comparisons, followed by manual review of possible title variants. A total of 207 duplicates were removed before screening; one additional title-variant duplicate identified during screening was reassigned to duplicate removal. Of 448 unique records screened, 278 were excluded, and 170 database reports were sought. Nine reports with substantive abstracts could not be retrieved.

Titles and abstracts were assessed against the written criteria, with uncertain records retained for full-text review. One review author resolved uncertainties and made all final eligibility decisions. The same reviewer assessed the full reports and verified source corrections and numerical transcriptions. Screening was performed by one reviewer and was not independently duplicated.

Data Charting, Critical Appraisal, and Synthesis

A structured form captured bibliographic details, study design, population or model, formulation, route, dose, and duration; vitamin B6 analyte, matrix, assay, threshold, and result; homocysteine, folate, vitamin B12, and related markers; clinical consequences; monitoring or supplementation; treatment response; limitations; and possible overlap. The form was iteratively calibrated and reapplied to the included reports. Reports from shared or sequential cohorts were linked and were not counted as independent populations.

No formal risk-of-bias or certainty assessment was undertaken. Design limitations, confounding, indirectness, selective testing, inconsistent assays, and cohort overlap were charted narratively. Results were synthesized by evidence level, route, clinical phenotype, and monitoring relevance. No meta-analysis or pooled prevalence was calculated because the studies differed substantially in selection, exposure, outcome definitions, testing, supplementation, and participant overlap.

Results

Selection and Characteristics of the Direct Evidence

**Figure 1:**
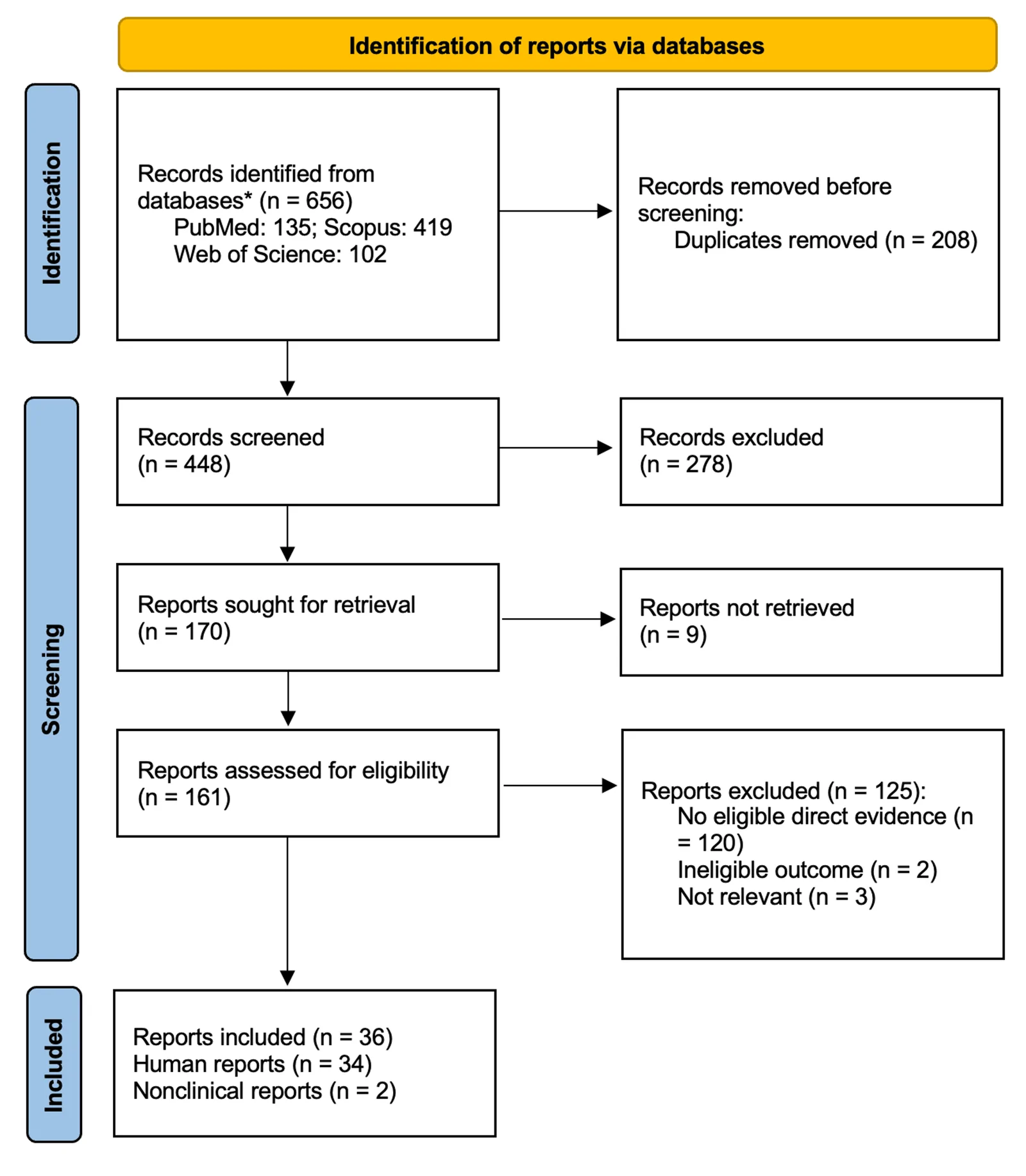
PRISMA-ScR flow diagram for identification, screening, retrieval, eligibility assessment, and focused inclusion of direct primary evidence. The searches identified 656 database records; after 208 duplicates were removed, 448 unique records were screened. Reports were sought for 170 records; nine were not retrieved, and 161 were assessed in full. Of these, 125 were excluded, and 36 met the narrowed content-based inclusion criteria. One title-variant duplicate was reassigned to duplicate removal during screening. PRISMA-ScR, Preferred Reporting Items for Systematic Reviews and Meta-Analyses extension for Scoping Reviews. Adapted by the authors from the PRISMA 2020 flow diagram template (Microsoft Word), distributed under the Creative Commons Attribution 4.0 International License (CC BY 4.0).

After 208 duplicates were removed, 448 records were screened, and 278 were excluded. Reports were sought for 170 database records; nine were not retrieved (Appendix B), and 161 were assessed in full. When the focused criteria were reapplied, 120 reports previously retained for indirect, supporting, or contextual use were assigned a single standardized primary exclusion reason: no eligible direct primary evidence for the narrowed review question. The remaining five full-text exclusions were assigned other primary reasons: an ineligible outcome in two reports and no relevance to the review question in three. In total, 125 reports were excluded after full-text assessment, and 36 were included (Figure 1).

The 36 included reports comprised 34 reports involving human participants and two nonclinical experimental reports. Human evidence consisted of 18 group-level studies and 16 case reports or case series. Publication years ranged from 1974 to 2026. Evidence covered oral therapy, levodopa/carbidopa intestinal gel, levodopa/entacapone/carbidopa intestinal gel, and subcutaneous foslevodopa/foscarbidopa. Vitamin B6 was represented by plasma or serum pyridoxal 5'-phosphate, pyridoxal, pyridoxine, an unspecified vitamin B6 analyte, functional kynurenine-pathway measures, or an adverse-event term. Assays, matrices, and reference intervals were not interchangeable. Twenty-five reports included either a funding statement or an explicit statement that no funding was received. Eight did not report funding, and funding status was unclear in three.

Direct Mechanistic Evidence

The structural study demonstrated a carbidopa-pyridoxal 5'-phosphate hydrazone within recombinant human dopa decarboxylase and characterized a catalytically relevant ketoenamine tautomer [5]. The animal toxicology study found biochemical and pathologic pyridoxine deficiency in dogs exposed to carbidopa, with prevention by coadministered pyridoxine [6]. These findings support target-level binding and the possibility of an in vivo deficiency phenotype, but neither study establishes the rate, reversibility, or systemic magnitude of pyridoxal 5'-phosphate loss at therapeutic exposure in humans.

Oral or Mixed Treatment Studies

Early functional studies were informative but inconsistent. A pooled carbidopa- or benserazide-treated group had altered kynurenine-pathway metabolites compatible with marginal impairment of pyridoxal 5'-phosphate-dependent metabolism, although circulating vitamin B6 was not measured and the two inhibitors were not separated [22]. In contrast, a Parkinson's disease comparator group receiving levodopa/carbidopa had normal plasma kynurenine and no significant acute change after a dose; direct plasma vitamin B6 activity was measured only in a comparator group without Parkinson’s disease [23]. An uncontrolled dietary and riboflavin intervention reported mean vitamin B6 values below its cited lower reference limit in both Parkinson's disease and dementia groups, without a significant between-group difference or exposure stratification [24].

More direct biomarker studies strengthened the association but did not establish causality. In 20 treated and 20 levodopa-naive patients, mean plasma pyridoxal 5'-phosphate was 78 versus 154 nmol/L, and homocysteine was 11.6 versus 8.8 micromol/L [7]. A 72-patient retrospective cohort found lower serum vitamin B6 in Parkinson's disease than in disease controls and an inverse adjusted association with total levodopa dose. The sign of a reported unadjusted correlation conflicted with the descending plot and the negative adjusted coefficient, and direct longitudinal vitamin B6 values were unavailable for the intestinal-gel subgroup [25]. In 262 levodopa-treated patients, serum pyridoxal did not differ with catechol-O-methyltransferase inhibitor use, while homocysteine was lower with inhibition; this supports treating homocysteine as a complementary rather than diagnostic vitamin B6 marker [26]. A postmortem preprint found lower brain pyridoxal 5'-phosphate in selected cognitive subgroups. All Parkinson's disease donors had received levodopa/carbidopa, but the postmortem design, limited dose and timing information, tissue calibration, and absence of an untreated Parkinson's disease comparator prevented a treatment-specific inference [27].

Intestinal Gel Studies

Intestinal-gel evidence included small cohorts, phase studies, extensions, and overlapping safety analyses. A 15-person cohort reported sensorimotor polyneuropathy in seven participants, and vitamin B6 deficiency in three, but the reported numbers of participants with vitamin B6 deficiency were internally inconsistent [28]. In a comparison of eight intestinal-gel and 13 oral patients, mean pyridoxine was 2.4 versus 5.5 micrograms/L, while the intestinal group received approximately 2.7 times the levodopa dose; pyridoxine correlated inversely with dose across groups [8]. Another 24-person study found low vitamin B6 in 19 participants, including all six receiving intestinal gel; symptoms compatible with neuropathy were not more frequent among vitamin B6-deficient participants, and fixed levodopa-to-carbidopa ratios prevented attribution to either component [9].

Prospective and extension data showed both low values and important ascertainment limitations. An open-label 39-person study recorded vitamin B6 deficiency in four participants and mild polyneuropathy events in three [29]. In a 262-person long-term extension, decreased vitamin B6 was coded in 58 participants and polyneuropathy-related events in 22; however, vitamin monitoring was introduced by protocol amendment, and supplementation was common [10]. A post hoc analysis reused this extension and other component trials and therefore did not provide an independent denominator [30]. In a 19-person prospective monitoring cohort, two incident neuropathies occurred among unsupplemented participants, and none among six nonrandomly supplemented participants, but the assay matrix changed during follow-up, and weight loss was also associated with neuropathy [31].

Longer observational studies did not resolve incidence. In an overlapping 16-hour and 24-hour intestinal-gel cohort, vitamin B6 deficiency was reported in 10 of 28 and nine of 35 observations, respectively, while neuropathy was less frequent during the later closely monitored phase [32]. A 10-year outpatient series found low vitamin B6 requiring supplementation in 20 of 34 assessed participants, but only a subset of the 80-patient cohort had reportable testing, and all low values were treated [33]. Conversely, a six-month levodopa/entacapone/carbidopa intestinal-gel study showed stable mean vitamin B6 and no diagnosed clinical polyneuropathy, although follow-up attrition, over-the-counter vitamins, lack of routine electrophysiology, and entacapone use limit this negative result [34].

Subcutaneous Treatment

The group-level subcutaneous evidence consisted of a small paired study of 18 patients starting foslevodopa/foscarbidopa. Among 12 with paired vitamin B6 measurements, mean concentration fell from 21.02 to 14.78 micrograms/L over a mean of 8.1 days, while homocysteine increased [11]. This within-person time course is compatible with treatment-related change but is too small and short to establish persistence, a clinical threshold, or a formulation-specific effect.

Clinical Consequences in Case Reports and Series

Seven reports described seizures or a mixed severe deficiency phenotype. Vitamin B6 was low or undetectable during oral, intestinal, or high-dose carbidopa/levodopa exposure, and seizures often persisted despite antiseizure medication. Rapid improvement after intravenous pyridoxine was reported in one case [35]. One additional report documented undetectable vitamin B6 after a carbidopa/levodopa dose increase, but the last seizure occurred one day before intravenous pyridoxine; the absence of recurrence during supplementation and after antiseizure-drug withdrawal is supportive but does not establish an immediate treatment response [36]. Several other cases also improved. Seizures stopped rapidly in some cases, whereas another case involved concurrent treatment changes [13,14,37]. One report documented severe hyperhomocysteinemia, profound pyridoxal 5'-phosphate deficiency, neuropathy, anemia, and seizure with improvement after vitamin replacement and dose reduction [38]. Another patient with intestinal gel exposure and profound deficiency died after delayed diagnosis despite treatment, illustrating that a temporal association and late rescue do not guarantee reversibility [39].

Seven reports focused on polyneuropathy. Four oral cases had documented deficiency, with biochemical correction and variable symptom improvement after supplementation [40]. Intestinal gel cases frequently combined low vitamin B6 with low folate or vitamin B12, high homocysteine, weight loss, diabetes, or pre-existing vulnerability [15,16,41]. Two inflammatory-appearing cases improved only after withdrawal of intestinal gel, despite vitamin replacement and immunotherapy, making an isolated deficiency mechanism uncertain [42]. With subcutaneous foslevodopa/foscarbidopa, painful axonal neuropathy improved after supplementation in one report without stopping infusion [17], while a case series of four patients showed biochemical and electrophysiologic deterioration against a background of baseline neuropathy or metabolic abnormalities [12].

Two reports described severe anemia. One patient developed transfusion-dependent microcytic anemia during chronic oral therapy and recovered after pyridoxine replacement [18]. Another developed severe sideroblastic-pattern anemia during intestinal gel and oral therapy, with very low vitamin B6 and improvement after pyridoxal phosphate while dopaminergic treatment continued [19]. These responses are compatible with impaired pyridoxal 5'-phosphate-dependent heme synthesis, but two case reports cannot establish frequency or exclude all alternative causes.

Monitoring and Supplementation

No randomized trial evaluated a monitoring schedule or a prophylactic vitamin B6 dose. The most direct prospective monitoring study included 19 patients and was nonrandomized [31]. Its investigators proposed assessment before intestinal-gel initiation, at six and 12 months, and annually thereafter, together with routine B-complex supplementation; neither the schedule nor prophylaxis was comparatively evaluated. Long-term cohorts treated low values as they were discovered, making the observed outcomes inseparable from surveillance and supplementation [10,33]. Case reports provide a rationale for considering vitamin B6 testing in patients with new seizures, neuropathic symptoms, unexplained microcytic anemia, weight loss, or high and escalating exposure, but replacement was often combined with folate, vitamin B12, nutritional support, antiseizure therapy, or dose modification [15,17,19,35,38,40].

**Figure 2:**
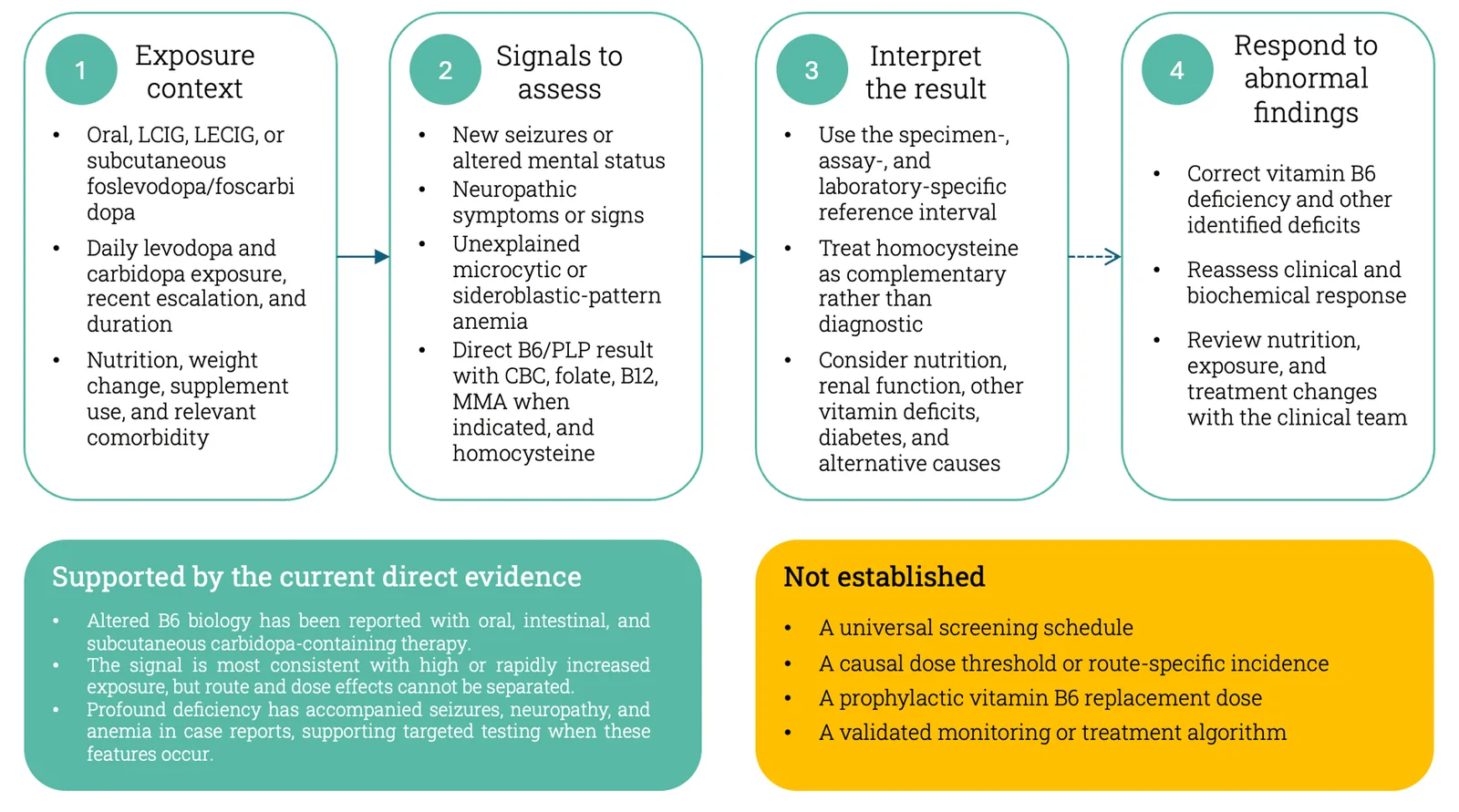
Context-sensitive assessment pathway for suspected vitamin B6 depletion during carbidopa/levodopa therapy. The four stages summarize exposure context, biochemical and clinical signals, interpretation of vitamin B6 status, and monitored response to a documented abnormality. A solid connector denotes an observed association or measured signal; dashed connectors denote evidence-informed but unvalidated steps. The lower panels distinguish supported elements from unresolved decisions. Testing should use the laboratory-specific specimen, assay, and reference interval. This is an interpretation aid, not a validated schedule, algorithm, or clinical guideline. Solid arrow: evidence-supported assessment step; Dashed arrow: evidence-informed but unvalidated response step B6, vitamin B6; B12, vitamin B12; CBC, complete blood count; LCIG, levodopa/carbidopa intestinal gel; LECIG, levodopa/entacapone/carbidopa intestinal gel; MMA, methylmalonic acid; PLP, pyridoxal 5′-phosphate. Figure created by the authors using Microsoft PowerPoint (Microsoft Corporation, Redmond, WA).

Direct measurements of vitamin B6 or pyridoxal 5'-phosphate should, therefore, be interpreted in relation to the specimen, assay, and laboratory-specific reference interval. Homocysteine can provide metabolic context but is influenced by folate, vitamin B12, renal function, age, diet, and catechol-O-methyltransferase inhibition [26]. A cautious assessment framework may combine direct vitamin B6 testing with folate and vitamin B12 assessment, functional cobalamin markers when indicated, a complete blood count with red-cell indices, nutritional and weight assessment, documentation of supplement use, and neurologic examination. Electrophysiology may be added when neuropathy is suspected (Figure 2). This framework summarizes the reviewed evidence and is not a validated schedule or clinical guideline.

Cohort Overlap and Absence of Pooled Estimates

Several publications represented linked populations. The 262-person extension was summarized again in the post hoc analysis, and the 39-person phase study was another component of that analysis [10,29,30]. The 16-hour and 24-hour observations reused participants [32], and the same center may have contributed to an earlier 15-person cohort [28]. Detailed cases were nested within the oral-versus-intestinal comparison [8], and other single-center series may also overlap. Report counts consequently describe the literature, not unique participants. No cross-report denominator, prevalence estimate, or route comparison was calculated.

Table 1 summarizes the design, sample, population or model, exposure, vitamin B6 assessment, and key findings of every included report. The entries were checked against the assessed full reports; linked cohorts remain separate reports and may contain overlapping participants.

**Table 1:** Characteristics and key findings of all 36 included reports. Each row represents a separate included report, but some reports contain overlapping participant populations. The cohorts reported by Fernandez et al. [10] and Standaert et al. [29] were also included in the post hoc cross-study analysis by Boyd et al. [30]. Eleven of the 15 participants reported by Chang et al. [28] also contributed to a dataset analyzed by Boyd et al. [30], and the 16-hour and 24-hour groups in Morales-Briceño et al. [32] represent overlapping treatment phases. Sample sizes should therefore not be summed across rows. “Not reported” means that the source did not provide the relevant information. B6, vitamin B6; BMRC, British Medical Research Council; COMT-I, catechol-O-methyltransferase inhibitor; DDC, dopa decarboxylase; DDCI, dopa decarboxylase inhibitor; EEG, electroencephalography; ENMG, electroneuromyography; FDR, false discovery rate; HPLC, high-performance liquid chromatography; IVIg, intravenous immunoglobulin; LCIG, levodopa/carbidopa intestinal gel; LECIG, levodopa/entacapone/carbidopa intestinal gel; LEDD, levodopa-equivalent daily dose; LOQ, limit of quantification; MedDRA, Medical Dictionary for Regulatory Activities; OLA, open-label extension; OLS, open-label study; P3b, phase 3b; PD, Parkinson's disease; PEG-J, percutaneous endoscopic gastrostomy with jejunal extension; PLP, pyridoxal 5'-phosphate.

Reference	Study design	Sample size	Study population/model	Exposure/route	B6/PLP assessment	Key findings
Baine et al. [5]	Experimental structural and kinetic biochemistry study	Not applicable (enzyme preparations)	Recombinant human DOPA decarboxylase and bacterial lysine decarboxylase	Carbidopa and synthetic α-hydrazino inhibitors	Direct PLP-hydrazone tautomer, binding and enzyme-inhibition assessment	Kinetics, spectroscopy and the human DDC-carbidopa crystal structure support formation of the catalytically relevant ketoenamine PLP hydrazone.
Zwickey et al. [6]	Preclinical animal toxicology study	B6-focused dog experiments: n=4/group (two male, two female); rat n=10-140/group and rhesus monkey n=2-12/group in subacute/chronic studies; acute LD50 work also included mice and rabbits	Dogs, rats, and rhesus monkeys; mice and rabbits in acute LD50 studies	Oral carbidopa alone; oral carbidopa/levodopa combinations; concurrent oral pyridoxine prevention in dogs	Longitudinal plasma pyridoxine, 24-hour urinary xanthurenic acid after an oral tryptophan challenge, and clinical/pathologic deficiency findings	Carbidopa caused biochemical and pathologic pyridoxine deficiency in dogs; concurrent pyridoxine attenuated lesions and prevented nerve lesions. No inferential p-values were reported.
Miller et al. [7]	Cross-sectional controlled clinical study	n=20 treated; n=20 levodopa-naive	Idiopathic PD treated with oral levodopa/carbidopa versus levodopa-naive PD	Standard oral levodopa/carbidopa	Plasma PLP: mean 78.0 versus 154 nmol/L (P=0.008), treated versus levodopa-naive.	Treated participants had lower PLP and higher homocysteine; within the treated group, homocysteine was inversely associated with PLP (R²=0.342; p=0.007), folate, and B12.
Loens et al. [8]	Retrospective comparative clinical study with nested cases	Laboratory cohort: LCIG n=8, oral n=13; neurography available in LCIG 6/8 and oral 11/13	Advanced idiopathic PD treated with LCIG or oral levodopa/carbidopa	LCIG versus oral levodopa/carbidopa	Plasma pyridoxine 2.4±1.4 versus 5.5±3.5 μg/L, LCIG versus oral therapy (P=0.01); inverse correlation with levodopa daily dose (Spearman r=-0.49, P=0.02)	Among neurographed participants, neuropathy occurred in six out of six LCIG cases and eight out of 11 oral therapy patients and was generally more extensive/severe with LCIG. The selected retrospective sample and large dose difference preclude attribution to route.
Rojo-Sebastián et al. [9]	Retrospective cross-sectional study	n=24 (oral n=18; LCIG n=6)	PD treated with levodopa/carbidopa for at least 3 years	Oral levodopa/carbidopa or LCIG	Fasting plasma B6 by ELISA (reference 23-172 nmol/L; LOQ 9 nmol/L): low in 19/24 (79.2%), including 6/6 LCIG and 13/18 oral; 9/24 were below LOQ. Median LCIG 9 versus oral 18.8 nmol/L (P=0.03).	Pearson correlations with prior-year cumulative carbidopa and levodopa doses were r=-0.36 and r=-0.33; exact P-values were not reported.
Fernandez et al. [10]	Prospective multinational open-label phase 3 extension	n=262; paired B6 values n=119	Advanced PD continuing from previous phase 3 LCIG studies	LCIG via PEG-J into the jejunum for 16 hours/day; oral levodopa/carbidopa permitted at bedtime or overnight	MedDRA-coded 'vitamin B6 decreased' in 58/262 (22%). In 119 paired nonfasting samples, mean B6 was 70.2±98.5 nmol/L at extension entry and 94.6±119.3 at endpoint; entry followed 52-74 weeks of prior LCIG. Supplementation was discretionary, and its overall prevalence was not reported.	Polyneuropathy-related adverse events occurred in 22/262 (8%); 19/22 affected participants received vitamin supplementation. Nonfasting testing, supplementation, and the absence of true pretreatment values prevented causal attribution.
Shaghaghi-Zadeh et al. [11]	Retrospective within-person research letter	n=18; paired B6 values n=12	Advanced PD starting foslevodopa/foscarbidopa	Continuous subcutaneous foslevodopa/foscarbidopa initiated during inpatient titration	Mean B6 fell from 21.02 to 14.78 micrograms/L over about 8 days (P=0.012).	Short-term within-person fall in B6 with a rise in homocysteine; folate and B12 did not change.
Vial-Daragon et al. [12]	Four-case series	n=4	PD with baseline subclinical neuropathy or biochemical vulnerability	Continuous subcutaneous foslevodopa/foscarbidopa	Serum B6 (reference 35-110 nmol/L): baseline 39.1, 30.9, 23.7, and 52.9; follow-up after symptom emergence 27.2, 18.2, 26.8, and 23.7 nmol/L. Follow-up sampling did not uniformly coincide with symptom onset.	Symptoms began at 68, 127, 60, and 90 days, with clinical and electrophysiological deterioration in all four patients. After withdrawal, case 1 had normalized biomarkers without clinical/ENMG recovery at six months; case 2 had normalized biomarkers and stable symptoms at three months but no repeat ENMG. Marked B6 oversupplementation may have contributed to worsening ataxia in case 3. Causality was not established.
Modica et al. [13]	Case report	n=1	An 83-year-old woman with PD	High-dose oral carbidopa/levodopa (2,100 mg levodopa/day; LEDD 2,475 mg), with gastroparesis, 9-kg weight loss/poor intake, and dietary B6 avoidance	Serum pyridoxine <5 nmol/L (reference 20-125 nmol/L); assay not reported	Clinical seizures ceased after IV lorazepam and levetiracetam escalation, but EEG still showed electrographic seizures and frequent multifocal discharges. IV pyridoxine produced immediate mental-status improvement, resolution of electrographic seizures, and fewer epileptiform discharges. The authors considered the deficiency multifactorial; later death followed failed extubation from severe laryngeal stenosis.
Skodda and Müller [14]	Case report	n=1	A 74-year-old woman with PD	LCIG plus nighttime oral levodopa/carbidopa	Serum 2.7 μg/L; CSF 1.3 μg/L	Refractory focal/nonconvulsive status epilepticus. Seizures ceased within two days; serum B6 increased to 23.3 μg/L, and homocysteine decreased to 18.54 μmol/L.
Klostermann et al. [15]	Two-case series nested in a 20-patient LCIG center cohort	n=2	PD patients who developed severe neuropathy after LCIG initiation	LCIG	2.5 and 2.2 μg/L	Severe axonal sensorimotor neuropathy. Weakness improved slowly; sensory symptoms largely persisted, and electrophysiology recovered only partly.
Urban et al. [16]	Two-case series	n=2	Advanced PD with neuropathy after 13 months of LCIG	Continuous intestinal levodopa/carbidopa gel monotherapy	3.0 and 3.6 μg/L	Biopsy-confirmed axonal sensorimotor neuropathy. Neuropathy stabilized for 10 months.
Valli and Scheperjans [17]	Case report	n=1	71-year-old woman with advanced PD	Continuous subcutaneous foslevodopa/foscarbidopa	41 nmol/L	Severe painful predominantly sensory axonal neuropathy. Improvement within two weeks; at two months B6 was 184 nmol/L and homocysteine 21 μmol/L.
Lee and Gastwirt [18]	Case report	n=1	A man in his early sixties with PD	Oral carbidopa/levodopa	1.2 μg/L	Transfusion-dependent severe microcytic anemia; hemoglobin 3.8 g/dL and MCV 72.5 fL. B6 normalized and hemoglobin exceeded 13 g/dL at one year, with no further transfusions.
Yasuda et al. [19]	Case report	n=1	A 75-year-old man with PD	16-hour LCIG plus oral levodopa/carbidopa and entacapone	<2.0 ng/mL	Severe microcytic, reticulocytopenic anemia; hemoglobin 6.6 g/dL, MCV 76.8 fL, reticulocytes <0.1%. Reticulocyte and hemoglobin response within one week; at six months hemoglobin 11.7 g/dL and B6 199 ng/mL, without reported worsening of parkinsonism.
Bender et al. [22]	Cross-sectional clinical biochemical study	Controls n=32; bromocriptine n=7; levodopa n=10; levodopa/DDCI n=32	PD treatment groups and controls	Oral levodopa plus DDC inhibitor: carbidopa/levodopa n=26 and benserazide/levodopa n=6	Urinary kynurenine-pathway metabolites indicated altered PLP-dependent tryptophan metabolism; circulating B6 was not measured.	The metabolite pattern was compatible with altered PLP-dependent tryptophan metabolism and possible marginal B6 deficiency.
Sternberg et al. [23]	Clinical biochemical study with a PD comparator group	PD comparator: n=12 in Methods; n=10 in abstract	Sinemet-treated PD comparator group within a broader biochemical study	Oral carbidopa/levodopa	Plasma kynurenine was age-normal and unchanged two hours after levodopa/carbidopa; B6 activity was not measured in the PD group.	Negative evidence against universal readily detectable functional B6 depletion during standard oral carbidopa/levodopa therapy.
Coimbra and Junqueira [24]	Prospective uncontrolled dietary/riboflavin intervention with a baseline dementia comparison group	Baseline biochemistry: PD n=31, dementia-without-stroke comparison n=10; intervention analysis n=19 completers (12/31 excluded for non-completion/non-adherence)	PD and dementia-without-stroke comparison groups	Riboflavin 30 mg orally every 8 hours plus red-meat elimination, with usual PD medication continued; medication included oral levodopa/carbidopa or levodopa/benserazide and other agents, while 3/31 were newly diagnosed and untreated	Baseline fasting heparinized-plasma pyridoxine by HPLC: 25.3±6.1 nmol/L in PD versus 24.3±9.4 in the dementia comparison group, not significant (reference 28-210 nmol/L)	Neutral baseline finding: mean pyridoxine did not differ between groups and both means were slightly below the stated lower reference limit. DDCI exposure was not stratified, so the result does not isolate carbidopa.
Ikenaka et al. [25]	Retrospective biomarker cohort with nested LCIG and opicapone analyses	PD n=72; disease controls n=31; nested LCIG n=24; nested LCIG+opicapone n=12	PD and disease controls, with nested LCIG/opicapone analyses	Predominantly levodopa/carbidopa, with a small levodopa/benserazide subgroup; oral and LCIG exposure	Serum B6 measured cross-sectionally by HPLC; lower in PD than disease controls (P=0.0153) and inversely associated with levodopa dose. B6 was not measured longitudinally in the LCIG/opicapone analyses; AST, ALT, ALT/AST, and homocysteine were surrogate outcomes.	B6 correlated with AST (r=0.448), ALT (r=0.575), and ALT/AST (r=0.449), all P<0.001; albumin was positively associated with B6. The longitudinal LCIG/opicapone findings were based on surrogate markers rather than repeat B6 measurements.
Kamiyama et al. [26]	Prospective cross-sectional two-center study	n=262 (carbidopa n=219; benserazide n=43). Results prose reports COMT-I n=77, whereas Table 1 analyzes COMT-I n=74 and non-COMT-I n=188.	Japanese PD patients receiving oral levodopa/DDCI therapy	Oral levodopa/DDCI with or without entacapone or opicapone	Cross-sectional serum pyridoxal 6.80±5.03 ng/mL overall; COMT-I 6.37±3.96 versus non-COMT-I 6.96±5.40 (P=0.335). Assay and collection timing were not reported.	COMT-I use was associated with lower homocysteine (β=-0.166, P=0.00024) but not higher pyridoxal. The unresolved COMT-I denominator discrepancy and cross-sectional design limit inference.
Kalecký et al. [27]	Preprint; postmortem case-control biomarker study	Cortex: controls n=36, Alzheimer disease n=35, PD n=65; putamen subset n=68	Postmortem brain tissue from PD, Alzheimer disease, and control donors	All Parkinson donors had received levodopa/carbidopa; route not reported	Targeted LC-MS/MS of postmortem cortex/putamen PLP, normalized to control SD. Cortical PD dementia showed a strong trend rather than a clearly significant individual-group reduction; significance strengthened when dementia groups were combined. Putaminal PD-MCI had lower PLP (β=-0.98 SD, P=0.006, FDR=0.026).	Human tissue findings support altered brain B6 biology in selected cognitively impaired PD subgroups, but results varied by brain region/subgroup and do not isolate carbidopa exposure.
Chang et al. [28]	Prospective open-label 12-month single-center cohort	First 15 consecutive patients: 4 compassionate-access and 11 phase III NCT00335153 participants	Advanced PD treated with LCIG	Intraduodenal LCIG up to 16 hours/day; immediate-release oral levodopa/carbidopa permitted at night	B6 deficiency reported in 3 participants; analyte, specimen, value, threshold, timing, and assay were not reported. The source also reported B12 deficiency in 4 and both deficiencies in 2, without reconciling category overlap.	Sensorimotor polyneuropathy occurred in 7/15 (46.7%; the paper incorrectly labels this 40%), with mean onset 13±0.4 months. Eleven participants also contributed to a linked phase III dataset.
Standaert et al. [29]	Open-label phase 3b study	Enrolled/safety n=39; successful PEG-J and LCIG/efficacy n=38; completed n=28	Advanced PD treated with LCIG	LCIG	MedDRA-coded treatment-emergent adverse event 'vitamin B6 deficiency' in 4/39 (10%); no analyte, specimen, unit, value, threshold, timing, or assay was reported	Polyneuropathy-related adverse events occurred in 3/39 (8%); all were mild and non-serious, none caused discontinuation, and one patient had a pre-existing peripheral nerve disorder.
Boyd et al. [30]	Post hoc cross-study analysis of six open-label phase 3/3b studies	Six linked studies: n=30-324 per analysis set	Advanced PD participants in six linked open-label LCIG studies	LCIG daytime monotherapy or polytherapy, with variable nighttime oral therapy	Decreased B6 was coded in linked cohorts; examples were 6/30 versus 7/32 and 37/164 versus 21/98.	Descriptive B6 adverse-event frequencies did not establish a monotherapy-versus-polytherapy effect.
Pauls et al. [31]	Retrospective chart review of a routinely monitored longitudinal cohort	n=19 (supplemented n=6; unsupplemented n=13)	Advanced PD starting LCIG without baseline clinical/electrophysiologic neuropathy	LCIG with or without a B-complex from initiation	Both incident neuropathy cases had a relative B6 decline; assay-matrix change prevented valid quantitative comparison.	New neuropathy occurred in two of 19 cases, both unsupplemented; none of six supplemented participants developed neuropathy. In unsupplemented participants, homocysteine rose from 14.2 to 19.7 μmol/L (p=0.010).
Morales-Briceño et al. [32]	Retrospective single-center cohort/research letter	24-hour LCIG n=35; prior 16-hour data n=28	PD receiving 24-hour LCIG, mostly after 16-hour infusion	Overlapping 16-hour and 24-hour LCIG phases	B6 deficiency: 10/28 during 16-hour therapy and 9/35 during 24-hour therapy.	De novo neuropathy was reported in 6/28 (21%) in the 16-hour phase and 1/35 (3%) during 24-hour treatment (p=0.02).
Magliozzi et al. [33]	Long-term retrospective single-center cohort/brief report	n=80; reportable B6 assessment n=34	Consecutive PD patients receiving outpatient-initiated LCIG	LCIG: 14 used 24-hour infusion at latest follow-up	B6 was decreased and supplemented in 20/34 assessed patients (58.8%).	Mean vitamin concentrations were maintained in range with supplementation; reported polyneuropathy frequency was 5%.
Viljaharju et al. [34]	Retrospective longitudinal observational study	n=30; 6-month follow-up n=19; paired laboratory data n=16	Advanced PD starting LECIG	LECIG; six patients switched from LCIG	Mean B6 was 106 nmol/L at baseline and 111 nmol/L at 6 months.	Mean (SD) homocysteine decreased from 18 (6.3) to 15 (3.9) μmol/L, and no clinical polyneuropathy was diagnosed.
Castro-Vilanova et al. [35]	Case report/letter	n=1	A 76-year-old woman with advanced PD	Continuous enteral levodopa/carbidopa infusion	13.7 nmol/L	Daily focal/generalized seizure-like episodes despite levetiracetam and lacosamide. Complete cessation of episodes and withdrawal of antiseizure drugs; at day 20, B6 was 149.3 nmol/L and homocysteine 13.9 μmol/L.
Nathan et al. [36]	Case report	n=1	A 76-year-old man with PD	Oral carbidopa/levodopa	Undetectable	New-onset medically refractory seizures. The last seizure occurred one day before pyridoxine; EEG was normal at nine and 21 months, lacosamide was stopped after two years, and seizures did not recur.
Wise et al. [37]	Case report	n=1	A 78-year-old man with PD	High-dose oral carbidopa/levodopa	<1 μg/dL	Refractory seizures. No further seizures; at the time of publication, the patient remained on two antiseizure drugs and IV B6.
Bjørke-Monsen et al. [38]	Case report	n=1	A woman in her sixties with PD	Oral carbidopa/levodopa plus tolcapone	PLP 1.2 nmol/L; homocysteine 225.9 μmol/L	Generalized seizure, severe painful axonal neuropathy, anemia, infections, vomiting, and approximately 30-kg weight loss. By day 16, PLP was 59.0 nmol/L and homocysteine 8.3 μmol/L; pain, general status, and reticulocyte response improved.
Baviera-Muñoz et al. [39]	Case report	n=1	A 77-year-old man with advanced PD	LCIG	<3.50 μg/L	Refractory status epilepticus with advanced general deterioration. Status epilepticus did not improve; the patient died with pneumonia and advanced deterioration.
Déry et al. [40]	Four-case series	n=4	PD patients with neuropathic symptoms	High-dose oral levodopa/carbidopa	<10, 17, <10, and 14 nmol/L	Neuropathic symptoms with heterogeneous nerve-conduction confirmation. Symptoms resolved in cases 1 and 4, were unchanged in case 2, and were not followed in case 3; post-treatment B6 was 831, 140, not reported, and 60 nmol/L.
Ueno et al. [41]	Case report	n=1	A 39-year-old man with PD and newly diagnosed diabetes	LCIG	Pyridoxal <2.0 ng/mL (reference 6.0-40.0); specimen and assay not reported	Subacute axonal sensory neuropathy. Laboratory normalization and symptomatic improvement over approximately two months; later LCIG reintroduction reportedly did not exacerbate symptoms.
Galazky et al. [42]	Two-case series	n=2	Two women with PD	LCIG	Patient 1: 2 ng/mL; patient 2: Not reported	Both patients progressed to severe tetraparesis despite IVIg; patient 1 additionally received methylprednisolone, plasmapheresis, and B-vitamin substitution. After LCIG withdrawal, both stabilized and then slowly partially recovered over three months, with residual BMRC grade-4 tetraparesis.

Discussion

Principal Interpretation

The direct evidence supports a coherent but incomplete chain from carbidopa/levodopa exposure to altered vitamin B6 biology. Carbidopa-pyridoxal 5'-phosphate binding is established at the target enzyme, and a deficiency phenotype can occur in dogs under high-dose carbidopa exposure. In humans, lower circulating vitamin B6 measures have been reported during oral, intestinal, and subcutaneous treatment, with the most consistent signals at high doses or after rapid escalation. However, no human study measured whole-body pyridoxal 5'-phosphate balance, separated the effects of levodopa from carbidopa, or isolated route from dose, disease severity, nutrition, and supplementation.

The evidence does not support a single prevalence estimate. Group-level studies differed in the vitamin B6 vitamer, specimen, assay, threshold, timing, and completeness of testing. Some studies used an adverse-event term without a reported concentration, while others measured functional markers rather than direct vitamin B6. Supplementation during follow-up could produce both low and high measurements in the same cohort. Neutral results, including normal functional markers during oral therapy and stable mean vitamin B6 during short-term levodopa/entacapone/carbidopa intestinal-gel treatment, argue against universal depletion.

Heterogeneity arose at several interconnected levels. Exposure ranged from intermittent oral treatment to 16- or 24-hour intestinal infusion and continuous subcutaneous infusion, while daily levodopa and carbidopa doses were variably reported and differed substantially where available; treatment duration and the timing of biomarker assessment also varied. Route was therefore confounded by dose, disease severity, and treatment duration. Nutritional reserve differed across reports: several involved weight loss, reduced intake, gastrointestinal dysfunction, or concurrent folate or vitamin B12 abnormalities, whereas these factors were inconsistently assessed in cohort studies. Vitamin supplementation was absent, discretionary, protocol-driven, or already established and could therefore attenuate biochemical changes or introduce confounding by indication. Studies also differed in the measured vitamin B6 vitamer, specimen, assay, threshold, and use of direct measurements, functional markers, or adverse-event codes. These differences limit direct comparisons and may partly explain the coexistence of marked deficiencies in selected reports and neutral group-level findings in other cohorts.

From Target-Level Chemistry to Systemic Deficiency

Target inhibition and systemic vitamin depletion are related but distinct propositions. Formation of a stable carbidopa-pyridoxal 5'-phosphate hydrazone within human dopa decarboxylase establishes how carbidopa inactivates a pyridoxal 5'-phosphate-dependent enzyme [5]. It does not by itself quantify removal of vitamin B6 from circulating, hepatic, neural, or other intracellular pools. Whole-body status also depends on dietary intake, intestinal absorption, hepatic interconversion of vitamers, protein binding, tissue redistribution, turnover, and excretion. None of the human studies measured these processes together or demonstrated a mass balance showing that therapeutic carbidopa exposure causes a net systemic loss of pyridoxal 5'-phosphate.

**Figure 3:**
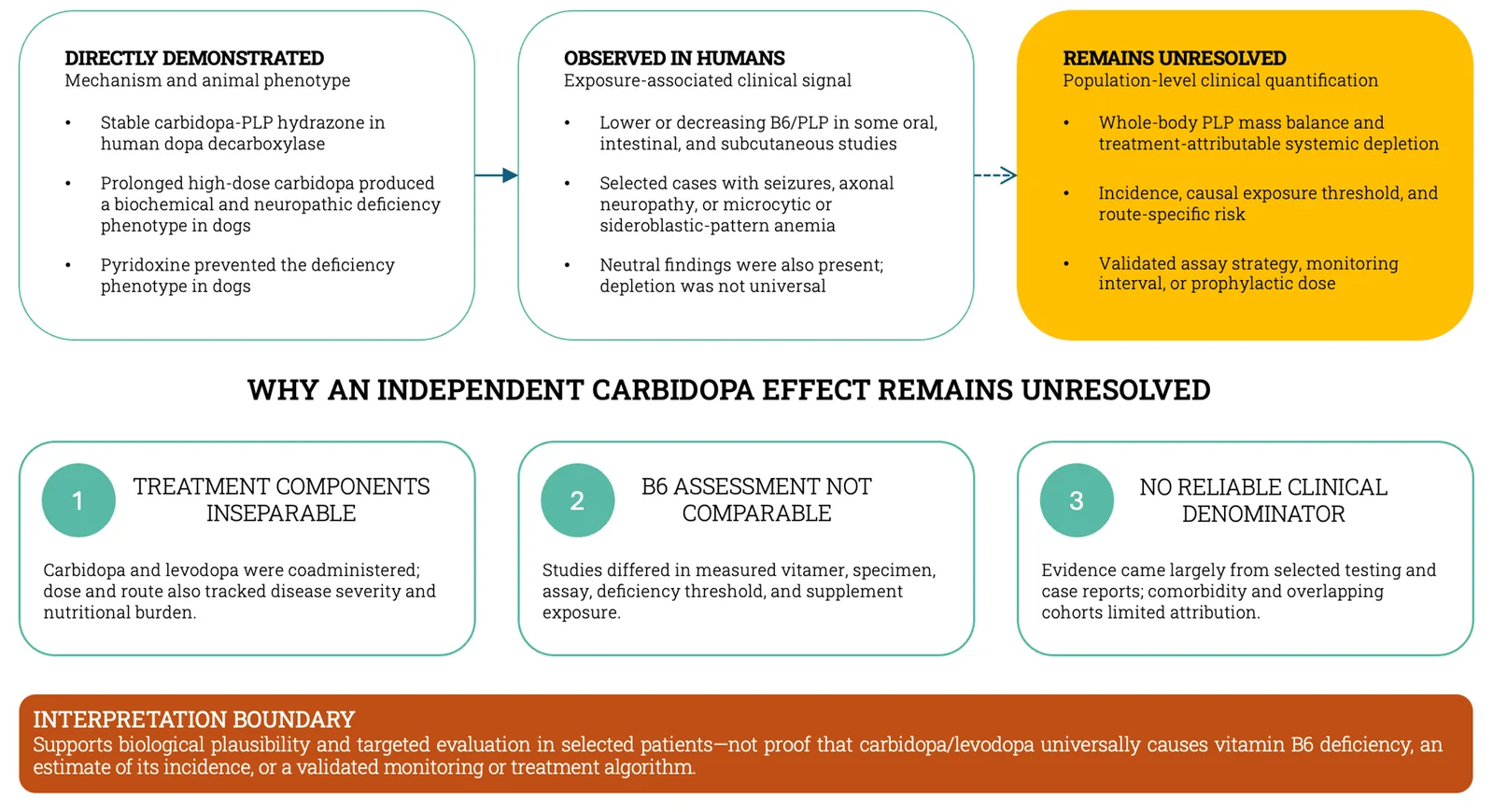
Evidence chain and inference boundaries for possible vitamin B6 depletion during carbidopa/levodopa therapy The upper row moves from directly demonstrated mechanistic and nonclinical findings [5,6], through representative human biomarker and clinical observations [7,8,11,13,15,18,19], to unresolved population-level questions. The solid arrow denotes biological plausibility; the dashed arrow marks the unquantified transition to population-level clinical effects. The lower panel lists three barriers to estimating an independent carbidopa effect: inseparable treatment exposure, non-comparable vitamin B6 assessment, and no reliable clinical denominator. The solid blue arrow indicates a mechanistic or empirically observed link that supports biological plausibility. The dashed orange arrow indicates an inferential transition that has not been directly established by the available evidence. B6, vitamin B6; PLP, pyridoxal 5′-phosphate. Figure created by the authors using Microsoft PowerPoint (Microsoft Corporation, Redmond, WA, USA).

The dog toxicology experiment partially addresses this inferential gap because high-dose carbidopa produced a systemic biochemical and neuropathic deficiency phenotype that pyridoxine prevented [6]. Its strength is whole-organism reversibility; its limitation is uncertain transferability from prolonged high-dose carbidopa exposure in dogs to therapeutic human treatment. Human evidence provides complementary but less decisive links. Lower pyridoxal 5'-phosphate in treated than levodopa-naive patients supports association [7], while the early decline after foslevodopa/foscarbidopa initiation supports temporality [11]. Neither study separated the effects of levodopa and carbidopa or established that the measured change reflected clinically important depletion rather than redistribution, altered metabolism, or limited nutritional reserve. The resulting causal chain is therefore strongest for target binding and biological plausibility, intermediate for an exposure-associated biomarker signal, and weakest for the incidence, magnitude, and clinical consequences of systemic depletion at specific human doses (Figure 3).

Reconciling Positive, Neutral, and Apparently Discordant Findings

The positive and neutral studies should not be treated as simple replications that produced opposite answers. They often assessed different biological levels. Direct plasma pyridoxal 5'-phosphate was lower in one controlled comparison [7], whereas earlier studies assessed kynurenine-pathway metabolites as functional indicators and reported either marginal abnormalities in a mixed decarboxylase-inhibitor group or no significant abnormality in a levodopa/carbidopa comparator group [22,23]. Another uncontrolled intervention found mean vitamin B6 values below its stated reference range in both Parkinson's disease and dementia groups, without a treatment-stratified difference [24]. These findings may reflect differences in analyte, matrix, reference interval, exposure intensity, nutritional status, or statistical power rather than a single uniformly reproducible treatment effect.

Longitudinal infusion evidence contains similar ascertainment differences. The large intestinal-gel extension recorded decreased vitamin B6 in many participants, but biochemical monitoring was introduced after a protocol amendment, and supplementation became common [10]. By contrast, mean vitamin B6 remained stable during a smaller six-month levodopa/entacapone/carbidopa intestinal-gel study, although individual supplementation, attrition, and the limited sample constrained its ability to exclude clinically important changes in susceptible patients [34]. Catechol-O-methyltransferase inhibition was associated with lower homocysteine without a corresponding difference in pyridoxal, further showing that related one-carbon biomarkers do not necessarily move together [26]. Neutral findings therefore narrow the hypothesis: they argue against inevitable depletion in every exposed patient, but they do not exclude dose-dependent or susceptibility-dependent depletion in a subgroup. Conversely, selected low-value cohorts and case reports cannot establish that such a subgroup is common.

Dose, Route, and Susceptibility

Intestinal gel cohorts generated much of the literature, but they also involved advanced disease and high daily exposure. In the most direct route comparison, the intestinal group received nearly three times the levodopa dose, and vitamin B6 correlated with dose across both groups [8]. Low values were also frequent during oral treatment [9], and biochemical change occurred soon after starting subcutaneous therapy [11]. An exclusively enteral malabsorption mechanism is therefore insufficient. Sustained exposure may interact with poor intake, weight loss, gastrointestinal dysfunction, renal disease, diabetes, baseline neuropathy, folate or vitamin B12 status, and individual vitamin reserve.

Future studies should report levodopa and carbidopa doses separately rather than relying only on levodopa-equivalent dose. They should also record formulation, treatment duration, catechol-O-methyltransferase inhibition, diet, supplements, body weight, albumin, renal function, inflammation, and disease severity. Without these variables, dose-response and route comparisons remain confounded by indication.

Clinical Meaning of Low Vitamin B6

Refractory seizures provided the clearest acute sentinel phenotype because several reports combined profound deficiency, resistance to antiseizure medication, and rapid response to pyridoxine. Even so, timing and cointerventions varied, and one critically ill patient did not recover after delayed replacement. Neuropathy was more frequent but less specific. Most reports described axonal injury with multiple nutritional or metabolic abnormalities, and established sensory deficits did not always reverse after biochemical correction. Inflammatory-appearing neuropathy sometimes required withdrawal of intestinal gel. The two anemia cases showed hematologic recovery after vitamin B6 replacement, including while dopaminergic treatment continued.

These patterns support active investigation of vitamin B6 deficiency in compatible presentations, not attribution of every neuropathy, seizure, or anemia to carbidopa. A low measured value is also not automatically proof of drug causation. Clinical interpretation should integrate exposure, timing, assay validity, diet, comorbidity, other vitamin markers, and response to a monitored intervention.

Neuropathy illustrates why attribution should remain graded rather than binary. Advanced Parkinson's disease populations already carry competing risks related to age, diabetes, renal impairment, reduced intake, weight loss, gastrointestinal dysfunction, immobility, and deficiencies of folate or vitamin B12. Several intestinal-gel and oral cases combined low vitamin B6 with hyperhomocysteinemia, other vitamin abnormalities, diabetes, or pre-existing neurologic vulnerability [15,16,41]. In the subcutaneous series, baseline electrophysiologic or metabolic abnormalities preceded treatment in some patients, making subsequent deterioration compatible with aggravation of an existing process rather than a wholly new drug-induced neuropathy [12]. The small prospective intestinal-gel cohort observed incident neuropathy only among unsupplemented participants, but supplementation was not randomized, and weight loss was also associated with neuropathy [31]. Inflammatory-appearing cases that improved only after intestinal-gel withdrawal despite vitamin replacement are also inconsistent with an isolated nutritional explanation [42].

Response to supplementation also requires cautious interpretation. Biochemical correction can occur more rapidly than recovery of established axonal injury, so persistent symptoms do not necessarily refute a contribution from deficiency. Conversely, improvement after supplementation is not definitive when folate, vitamin B12, nutritional support, dose modification, antiseizure drugs, or rehabilitation are introduced simultaneously. A clinically useful synthesis should therefore distinguish deficiency as the likely primary mechanism, a plausible contributing factor, a coincidental laboratory finding, or an uncertain association. The available reports support all four categories and do not justify collapsing them into a single carbidopa-induced neuropathy entity.

Implications for Monitoring

The included evidence cannot define a universal testing interval, deficiency threshold across laboratories, or preventive dose. It supports considering a direct vitamin B6 measure when exposure is high or rapidly increasing, when continuous infusion is initiated, or when a patient develops weight loss, neuropathic symptoms, unexplained microcytic anemia, or new seizures. A baseline value before major escalation may be informative because several infusion cases had pre-existing biochemical or electrophysiologic abnormalities [12]. Periodic follow-up may be considered in high-risk contexts, but neither its clinical benefit nor an interval has been established in comparative studies.

Homocysteine should not substitute for vitamin B6 measurement. It is generated through levodopa methylation but also varies with folate, vitamin B12, renal function, age, diet, genotype, and catechol-O-methyltransferase inhibition. When supplementation is used for documented deficiency, biochemical and clinical response should be reassessed because the preventive target and long-term dose have not been tested in this population. Marked vitamin B6 oversupplementation accompanied worsening ataxia in one included case, reinforcing the need to avoid unmonitored high-dose treatment [12]. The present review supports monitored correction of deficiency, not universal pharmacologic-dose pyridoxine prophylaxis.

Outside the set of 36 included reports, the March 2026 US FDA Drug Safety Communication required warnings about vitamin B6 deficiency and associated seizures for carbidopa/levodopa products [21]. The underlying safety review identified 14 deficiency-associated seizure cases: 13 postmarketing reports and one literature case. All involved levodopa doses above 1,000 mg/day; doses above 1,500 mg/day were associated with a shorter interval to documented deficiency, and latency ranged from 23 to 132 months across oral and enteral formulations. Seizures resolved in all nine patients reported to have received vitamin B6, while two deaths occurred. The communication advises evaluating vitamin B6 before treatment, periodically during treatment, and when deficiency symptoms emerge, with supplementation as necessary. Because no exposed denominator was available and overlap with published cases could not be resolved at report level, these pharmacovigilance cases were not pooled with the 36 included reports and cannot yield an incidence estimate. The regulatory action strengthens the clinical rationale for surveillance but does not validate an assay, threshold, monitoring interval, or replacement dose.

The assessment framework in Figure 2 consequently separates three decisions that are often conflated: whether testing is warranted, how a result should be interpreted, and whether intervention is indicated. Baseline testing before continuous therapy or a major dose escalation may reveal pre-existing vulnerability and provide a within-person comparator, but its incremental clinical benefit has not been tested. During treatment, high or rapidly increasing exposure, weight loss, reduced intake, neuropathic symptoms, new seizures, or unexplained microcytosis can increase the rationale for reassessment. Interpretation should consider the measured vitamer, specimen, assay, laboratory reference interval, supplement timing, and concurrent folate, vitamin B12, renal, hematologic, and nutritional findings. A trend within the same assay may be informative, but no included study established a clinically meaningful rate of decline.

Intervention is a separate step. The evidence supports monitored correction of documented deficiency and evaluation of associated clinical abnormalities; it does not establish routine pharmacologic-dose prophylaxis for every treated patient. Normalization of a laboratory value, improvement of symptoms, and prevention of future complications are distinct outcomes and may occur on different timelines. The FDA communication establishes a regulatory basis for baseline and periodic evaluation, but the reviewed studies do not define a universal monitoring interval or replacement dose. Figure 2 should therefore be read as a structured interpretation aid that identifies evidence-supported considerations and unresolved decisions, not as a validated algorithm, professional-society guideline, or substitute for individualized clinical judgment.

Research Priorities

The key need is prospective, exposure-resolved human evidence. Multicenter inception cohorts should measure vitamin B6 before treatment initiation or major escalation and at prespecified early and longitudinal time points with harmonized assays. Within-person studies of dose escalation and route switching could help separate dose from formulation. Mechanistic studies should quantify free and protein-bound pyridoxal 5'-phosphate pools, carbidopa-pyridoxal 5'-phosphate adduct kinetics, tissue distribution, reversibility, and excretion at therapeutic exposure. Biomarker-defined supplementation trials should compare doses, time to correction, neurologic and hematologic outcomes, motor response, and safety. Pharmacovigilance studies require exposed denominators and deduplication against published cases.

Limitations

The focused eligibility rule was developed after completion of a broader evidence map, creating a risk of post hoc selection even though it was translated into content-based criteria and reapplied to every retrieved report. The search was limited to PubMed, Scopus, and Web of Science. No separate systematic search of trial registries, dissertation repositories, preprint servers, or regulatory websites was performed. Nine reports with substantive abstracts were not retrieved; seven were published before 2000, including six from the 1970s, whereas the remaining two were published in 2005 and 2009. Records without an abstract were excluded during screening unless sufficient information could be obtained from an accessible full text; this may have underrepresented older and non-English-language literature. Because secondary reviews, regulatory guidance, and indirect pharmacology were intentionally excluded from the evidence set, this focused synthesis should not be interpreted as a comprehensive review of current product labeling or historical pyridoxine-levodopa pharmacology. The search was completed on July 21, 2026; reports indexed after that date were not captured.

Final screening and charting were performed by one review author without independent duplicate review. No protocol was prospectively registered or published, and no formal critical appraisal was performed. The absence of a prospectively registered or published protocol limited prospective methodological transparency and, together with the post hoc narrowing of the eligibility criteria, increased the possibility of selective methodological decisions. The direct evidence was dominated by small, retrospective, uncontrolled, or overlapping studies and case reports susceptible to selection bias. Vitamin B6 measurements were heterogeneous, supplementation was common, and exposure variables were often inseparable. One included report was a preprint. Publication bias is also possible because severe or unusual deficiency presentations are more likely to be reported than uneventful exposure or normal vitamin B6 findings. These limitations preclude causal attribution, valid incidence estimates, route-specific risk estimates, or dosing recommendations.

## Conclusions

Direct experimental evidence establishes carbidopa-pyridoxal 5'-phosphate binding, while human observational evidence supports lower vitamin B6 biomarker values during some carbidopa/levodopa exposures, particularly at high or rapidly increased doses. Severe deficiency can accompany seizures, axonal neuropathy, and microcytic or sideroblastic-pattern anemia, but susceptibility is heterogeneous and competing nutritional or metabolic factors are common. The evidence supports context-sensitive biochemical assessment and monitored correction of documented deficiency rather than reliance on homocysteine alone or universal high-dose supplementation. Prospective studies with harmonized assays and separately resolved levodopa and carbidopa exposure are needed before monitoring intervals, route-specific risk, deficiency thresholds, or preventive regimens can be defined.

Targeted vitamin B6 monitoring may be most informative in patients starting continuous intestinal or subcutaneous therapy, receiving high or rapidly increasing carbidopa/levodopa doses, or developing weight loss, reduced intake, neuropathic symptoms, unexplained microcytic or sideroblastic-pattern anemia, or new seizures. Current evidence supports monitored correction of documented deficiency but does not establish a universal monitoring interval or justify routine pharmacologic-dose pyridoxine supplementation for every treated patient.

## Appendices

Appendix A 

**Table 2:** Exact database search strategies used in the focused scoping review

Database	Exact search strategy
PubMed	( "Parkinson Disease"[Mesh] OR Parkinson*[Title/Abstract] ) AND ( "Levodopa"[Mesh] OR levodopa[Title/Abstract] OR "L-DOPA"[Title/Abstract] OR carbidopa[Title/Abstract] OR "carbidopa-levodopa"[Title/Abstract] OR "levodopa-carbidopa intestinal gel"[Title/Abstract] OR LCIG[Title/Abstract] OR foslevodopa[Title/Abstract] OR foscarbidopa[Title/Abstract] ) AND ( "Vitamin B 6"[Mesh] OR "vitamin B6"[Title/Abstract] OR "vitamin B-6"[Title/Abstract] OR pyridoxin*[Title/Abstract] OR "pyridoxal phosphate"[Title/Abstract] OR "pyridoxal 5-phosphate"[Title/Abstract] OR PLP[Title/Abstract] )
Scopus	TITLE-ABS-KEY ( Parkinson* AND ( levodopa OR "L-DOPA" OR carbidopa OR "carbidopa-levodopa" OR "levodopa-carbidopa intestinal gel" OR LCIG OR foslevodopa OR foscarbidopa ) AND ( "vitamin B6" OR "vitamin B-6" OR pyridoxin* OR "pyridoxal phosphate" OR "pyridoxal 5-phosphate" OR PLP ) AND ( deficien* OR deplet* OR neuropath* OR seizure* OR epilep* OR anemia OR anaemia OR homocystein* OR monitor* OR supplement* ) )
Web of Science Core Collection	TS=( Parkinson* AND ( levodopa OR "L-DOPA" OR carbidopa OR "carbidopa-levodopa" OR "levodopa-carbidopa intestinal gel" OR LCIG OR foslevodopa OR foscarbidopa ) AND ( "vitamin B6" OR "vitamin B-6" OR pyridoxin* OR "pyridoxal phosphate" OR "pyridoxal 5-phosphate" OR PLP ) AND ( deficien* OR deplet* OR neuropath* OR seizure* OR epilep* OR anemia OR anaemia OR homocystein* OR monitor* OR supplement* ) )

Appendix B 

**Table 3:** Reports sought but not retrieved *Abbreviations*: PMID, PubMed Unique Identifier; Scopus EID, Scopus Electronic Identifier.

Authors and year	Report title	Journal or source	Persistent identifier
Caceres AF; 1974	Results of treatment of Parkinson's disease with carbidopa and levodopa	Revista de la Facultad de Ciencias Medicas de la Universidad Nacional de Cordoba	PMID: 4619691
Miller JW, Thomas CA, Nadeau MR, et al.; 1997	Effect of L-Dopa administration on blood homocysteine concentration in Parkinson's patients	FASEB Journal	Scopus EID: 2-s2.0-33750189820
Joerg J, Kleine D; 1979	Risks of modern therapy for Parkinsonism, and their consequences	Nervenarzt	PMID: 424033
Ohmoto T, Kishikawa H; 1975	Levodopa plus a decarboxylase inhibitor (carbidopa) in parkinsonism: clinical effects and catecholamine metabolism (Japanese)	Brain and Nerve	Scopus EID: 2-s2.0-0016614122
Evtushenko SK; 1976	[Combined treatment of Parkinsonism with levopa, midantan and anticholinergic drugs]	Zh Nevropatol Psikhiatr Im S S Korsakova	PMID: 1015115
Hanzal F; 1976	[Treatment of Parkinson's syndrome with L-dopa and L-carbidopa (author's transl)]	MMW Munch Med Wochenschr	PMID: 818540
Bialecka M, Rabowski P, Honczarenko K, Roszmann A, Slawek J; 2009	Genetic and environmental factors for hyperhomocysteinaemia and its clinical implications in Parkinson's disease	Neurol Neurochir Pol	PMID: 19618311
Ebadi MS, Costa E; 1972	Role of vitamin B6 in neurobiology	Raven Press, New York	Scopus EID: 2-s2.0-0015485979
Gao YH; 2005	Levels of homocysteine in plasma associated with the levels of folic acid and vitamin B6 in patients with Parkinson disease treated with Madopar	Chinese Journal of Clinical Rehabilitation	Scopus EID: 2-s2.0-27644520259
